# Thanks to reviewers

**DOI:** 10.1007/s40037-016-0253-9

**Published:** 2016-02-08

**The Editorial Team would like to extend our gratitude to all reviewers for their time and advice that helped us to assess and improve the papers that were submitted to us in 2015. The reviewers’ critical and constructive feedback greatly helped to raise the journal’s quality.**

Azam Afzal

Coby Baane

Dorene Balmer

Glen Bandiera

Hamid Baradaran

Joanna Bates

Rohini Bhadre

Alan Bleakley

Peter Bloemendaal

Peter Boendermaker

Sanneke Bolhuis

Benno Bonke

Nicole Borges

Jan Borleffs

Paul Brand

Johan Bredée

Craig Brown

Ryan Brydges

Jamiu Busari

William Bynum

Diana Callender

Peter Cantillon

Saad Chahine

Ming-Ka Chan

Teresa Chan

Carrie Chen

Yan Chen

Jeffrey Cheung

Carlos Collares

Peter Coventry

Gerda Croiset

Mary E.W. Dankbaar

Peter de Jong

Benedictine De Winter

Ann Deketelaere

Friedo Dekker

Joke Denekens

Marja Dijksterhuis

Diana H.J.M. Dolmans

Tim Dornan

Jos Draaisma

Robbert Duvivier

Samuel Edelbring

Rachel Ellaway

Lia Fluit

Jacques Forest

Adrian Freeman

Jayne Garner

Trevor Gibbs

Marjan Govaerts

Esther Groot

Frans Grosfeld

Jose Hanham

Richard Hays

Harianne Hegge

Marjolein Heijne-Penninga

Marcus Henning

Karen Jerardi

Sundari Joseph

Rosaiah Kanaparthy

Jan Willem Kappelle

Brian Kelly

George Kim

Lars Konge

Jill Konkin

Wim Kremer

Frank Krings

Kulamakan Kulasegaram

Ayelet Kuper

Rashmi Kusurkar

Felicitas Lahner

Vicki leblanc

Janet Lefroy

Jimmie Leppink

Marcel Levi

Joule Li

Lorelei Lingard

Mario Maas

Anna MacLeod

Bente Malling

Katri Manninen

Nicole Mastenbroek

Danette Mckinley

Lindsay Melvin

Sonya Miller

Willemina Molenaar

Sandra Monteiro

Jolene Moore

Laura Naismith

Gita Negi

Laura Nimmon

Zineb Nouns

Arko Oderwald

Rebecca Olson

Lotte O’Neill

Maria Opfermann

Vimmi Passi

Dominique Piquette

Martien Quaak

Simon Riley

Clemens Rommers

Jerome Rotgans

Mirabelle Schaub-de Jong

Stefan Schauber

Felix Schmitz

Valerie Schulz

Lambert Schuwirth

Jonathan Sherbino

Ruth Sladek

Dominique Sluijsmans

Lesley Southgate

Annemarie Spruijt

Renee Stalmeijer

Fred Stevens

Andrea Stockl

Pia Strand

Roger Strasser

Javeed Sukhera

Walter Tavares

Edith ter Braak

Ian Thomas

Rene Tio

Andre Tricot

Sebastian Uijtdehaage

Jan van Dalen

Sjoukje Van den Broek

Cees van der Vleuten

Nynke van Dijk

Yvonne van Leeuwen

Paul Van Royen

Marta van Zanten

Shannon Venance

Kenneth Walker

Tineke Westerveld

Indah Widyahening

Kalman Winston

Leonard Witkamp

Yolande Witman

Sarah Wright

Sarah Yardley

